# A computational study on the spatial correlation of granule cell firing in the cerebellar cortex

**DOI:** 10.1186/1471-2202-13-S1-P153

**Published:** 2012-07-16

**Authors:** Daqing Guo, Mario Negrello, Erik De Schutter

**Affiliations:** 1 Computational Neuroscience Unit, Okinawa Institute of Science and Technology, Okinawa 904-0411, Japan; 2 Theoretical Neurobiology, University of Antwerp, B-2610 Antwerp, Belgium

## 

The cerebellum, also known as small brain, is a special region of the brain and plays critical roles in motor control. The granular layer, which harbors more than 95% of the cerebellar neurons (and more than 50% of the cells in the whole brain), is the input layer of the cerebellum, including two main neuron types: granule cells (GrCs) and Golgi cells (GoCs). From a functional perspective, the cerebral granular layer is responsible for receiving the mossy fiber (MF) inputs originally from sensory systems, cerebral cortex and spinal cord. These MF signals are then transformed and sent to the molecular layer, and eventually the Purkinje cells, to be further integrated to fine tune motor activities. Accordingly, elucidating the spatial/temporal dynamics of the granular layer is fundamental to understand the overall signal processing in the cerebellum. However, there remain many dynamical enigmas at the level of input integration relating to the spatial-temporal integration potentialities.

In order to identify them, we built a large-scale 3D network model of the granular layer. The model (in NEURON) has dimensions 1 × 1 × 0.2 mm3, and contains both GrCs and GoCs with realistic cell and synapse counts, and has connectivity tightly constrained by anatomical literature [1]. Both cells receive excitatory inputs from MFs, and had their dynamics modeled according to detailed models, published in [2,3]. In the network, the GrCs (single-compartment) excited GoCs (multi-compartment) via parallel fibers. GoCs were the only source of inhibition for their surrounding GrCs. There was no connection between different GrCs, and gap junction coupling between GoCs was not considered.

In this poster, we will present results on the properties of the spread of activity in the granular layer. In addition, we will discuss how the dynamical properties of MF inputs influence the spatial correlation of granule cell firing in the cerebellar cortex. Our results indicate that (1) the spatial correlation of granule cell firing is rather local, and (2) displays very short transient, and (3) exhibits a level of anisotropy. We hope that this work will contribute not only to the basic understanding of the spatial dynamics of the granule layer, but also to the capabilities of the cerebellum to integrate multimodal input.
